# Case Report: Experience in surgical management of vertebral posterior marginal osteophytes completely encasing the nerve root: a report of two cases

**DOI:** 10.3389/fsurg.2025.1618743

**Published:** 2025-11-14

**Authors:** Feng Li, Xingguo Tan, Mingjia Song, Lian Zhang, Songkai Li

**Affiliations:** 1 Department of Spinal Surgery, The 940th Hospital of the Joint Logistic Support Force of Chinese People’s Liberation Army, Lanzhou, China; 2 Department of Orthopedics, The 943rd Hospital of Joint Logistic Support Force of Chinese People’s Liberation Army, Wuwei, China; 3 First Clinical Medical School, Gansu University of Chinese Medicine, Lanzhou, China

**Keywords:** lumbar disc herniation, osteophytes, nerve root injury, case report, spinal canal decompression, discectomy

## Abstract

Posterior marginal osteophytes at the vertebral level are frequently observed in individuals with lumbar disc herniation (LDH); however, complete encasement of the nerve root by such osteophytes is exceedingly uncommon. We present two cases of surgical management of vertebral posterior marginal osteophytes completely encasing the nerve root. This report examined two cases of LDH accompanied by vertebral posterior marginal osteophytes completely encasing the nerve roots. Both patients underwent spinal canal decompression, discectomy, and intervertebral fusion. Postoperatively, varying degrees of nerve root injury were noted. By the 3-month follow-up, marked recovery in neurological function was achieved in both cases. The presence of vertebral posterior marginal osteophytes completely encasing the nerve root represents a notable risk factor for nerve root injury in LDH cases. Tailored surgical approaches are imperative to minimize complications and optimize patient outcomes.

## Introduction

Vertebral osteophyte formation represents a hallmark of intervertebral disc degeneration (1), with increased prevalence observed in individuals with lumbar disc herniation (LDH) (2). While anterior and lateral vertebral osteophytes generally do not cause nerve-root symptoms (3), intervertebral foraminal osteophytes frequently encase and compress nerve roots (4). In contrast, complete encasement of nerve roots by vertebral posterior marginal osteophytes remains an infrequent occurrence. The etiology of vertebral posterior marginal osteophytes encompasses posterior apophyseal ring separation, intervertebral disc calcification, ossification of the posterior longitudinal ligament, and vertebral posterior marginal hyperplasia (5–7). Nerve roots entirely encased and subjected to severe compression by osteophytes render conservative treatments largely ineffective. Optimal management necessitates meticulous nerve root exploration, decompression, lumbar discectomy, and interbody fusion (8, 9). The aberrant anatomical positioning of the nerve roots significantly heightens the risk of iatrogenic injury during surgical intervention. To date, no published literature addresses experience of surgical management in cases of LDH complicated by vertebral posterior marginal osteophytes that completely encase the nerve roots. This report presents two patients with LDH and vertebral posterior marginal osteophytes completely encasing the nerve roots, who experienced varying degrees of nerve root injury symptoms following surgical procedures, including spinal canal decompression, nerve root exploration and release, lumbar discectomy, interbody fusion, and fixation. By the 3-month follow-up, postoperative recovery of nerve root function was achieved in both cases. Written informed consent for publication of their clinical details and images was obtained from the patients.

## Case reports

### Case 1

**Figure 1:**
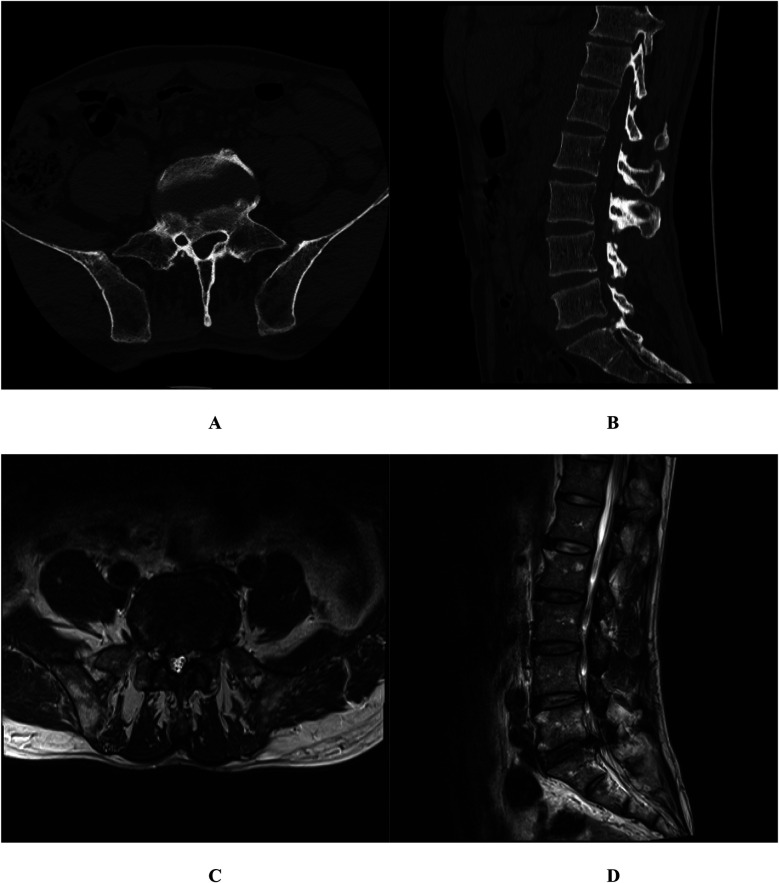
Case 1. Three-dimensional (3D) lumbar Computed Tomography (CT) demonstrated a posterior marginal separation of the S1 vertebra and a calcified protrusion of the L5-S1 intervertebral disc extending toward the right posterior region **(A,B)**; Lumbar Magnetic Resonance Imaging (MRI) confirmed an L5-S1 disc herniation **(C,D)**.

**Figure 2:**
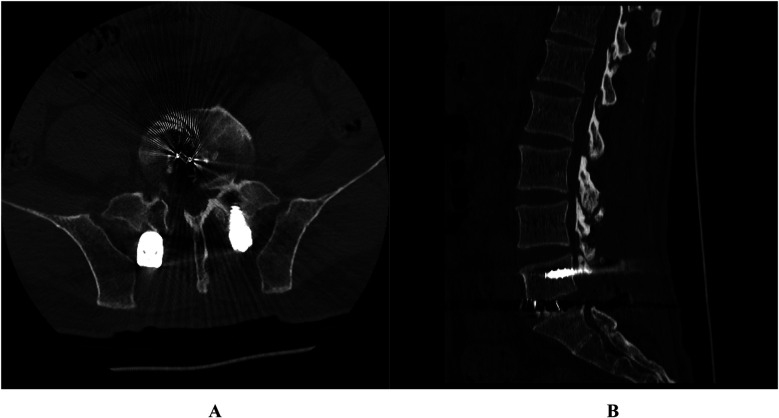
Case 1. Postoperative lumbar 3D CT showed that the vertebral posterior margin osteophytes were completely removed and the nerve roots were released. **(A)** Transverse image; **(B)** Lateral image.

A 52-year-old male with a 20-year history of smoking presented with a three-year history of persistent low back pain accompanied by right lower limb pain and numbness. Symptoms were localized to the right buttock, posterolateral thigh, calf, and sole. Physical examination revealed tenderness and percussion pain over the L4-S1 segment, diminished muscle tone in the right lower limb, and positive results in the straight leg raising and strengthening tests for the same limb. No significant abnormalities were noted in other physical evaluations. Three-dimensional (3D) lumbar Computed Tomography (CT) demonstrated a posterior marginal separation of the S1 vertebra and a calcified protrusion of the L5-S1 intervertebral disc extending toward the right posterior region (Figures 1A,B). Lumbar Magnetic Resonance Imaging (MRI) confirmed an L5-S1 disc herniation (Figures 1C,D). The diagnosis of LDH with radiculopathy was established. Preoperative pain and functional disability were assessed using the Visual Analog Scale (VAS) and Oswestry Disability Index (ODI), with scores of 5/10 and 72, respectively. Surgical intervention involved right hemilaminectomy, spinal canal decompression, nerve root exploration and release, lumbar discectomy, fusion cage implantation, bone graft, intervertebral fusion, and fixation (Figure 2). Intraoperatively, the posterior marginal osteophyte of the S1 vertebra was observed to displace the S1 traversing nerve root outward into the lateral recess. S1 nerve root was completely encased by a narrowed, bone tunnel-like structure at the upper edge of the S1 vertebra. Postoperatively, the patient exhibited plantar flexion weakness in the right lower limb, with triceps surae muscle strength graded at Medical Research Council (MRC) grade 3, while other muscle groups retained normal strength (grade 5). At the 3-month follow-up, the triceps surae muscle strength improved to MRC grade 4+. The VAS score improved to 1/10, and the ODI score significantly improved to 18, indicating minimal pain and mild disability. The overall outcome assessed by Macnab Criteria was rated as “good”.

### Case 2

**Figure 3:**
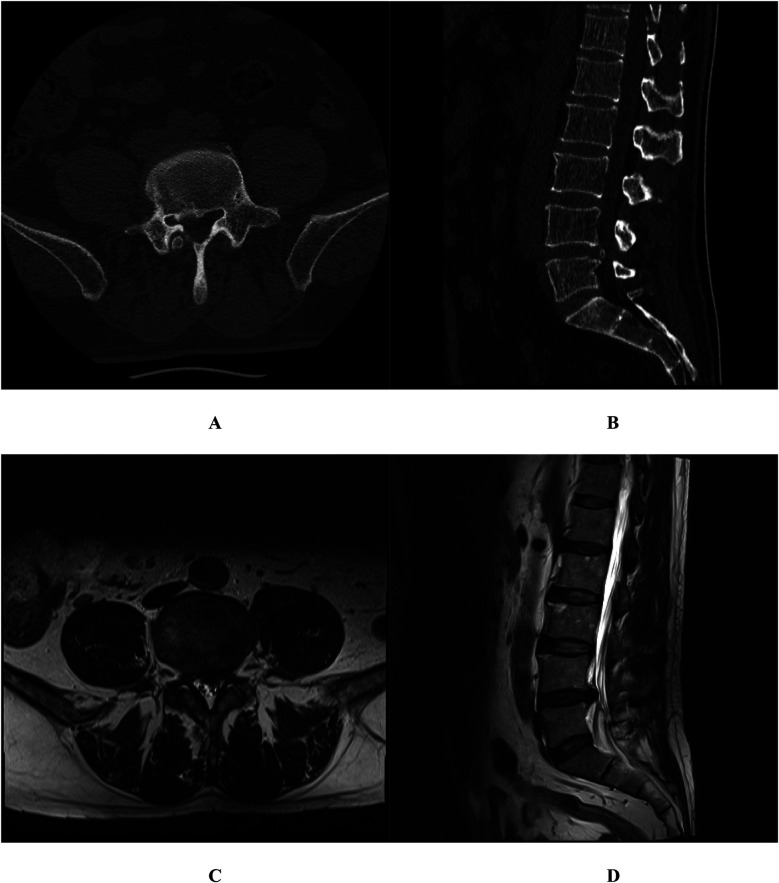
Case 2. Lumbar 3D CT identified a posterior marginal separation of the L5 vertebra and a calcified, right-posterior L4-5 intervertebral disc protrusion **(A,B)**; Lumbar MRI confirmed L4-5 intervertebral disc herniation and associated spinal canal stenosis **(C,D).**

**Figure 4:**
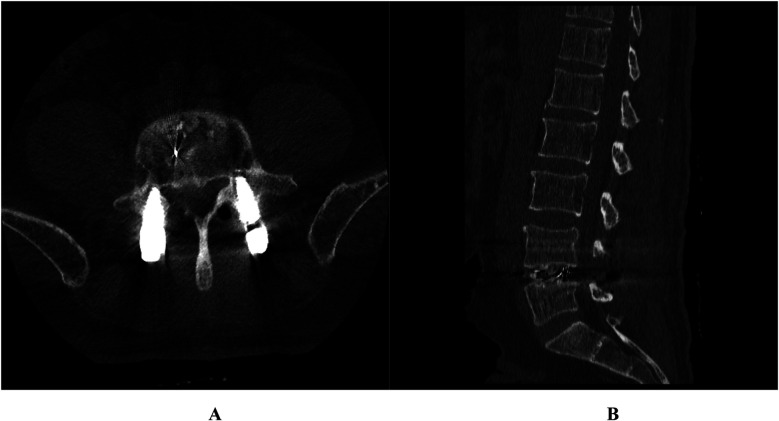
Case 2. Postoperative lumbar 3D CT showed that the vertebral posterior margin osteophytes were completely removed and the nerve roots were released **(A,B)**.

A 60-year-old male with a 40-year smoking history and a 1-year history of hypertension presented with a 6-year history of low back pain, recently exacerbated by pain and numbness in the left lower limb for 3 months. He previously experienced intermittent pain and numbness in the right lower limb, which resolved spontaneously. Current symptoms include pain and numbness in the left buttock, posterior and lateral thigh, and lateral calf, accompanied by intermittent claudication. Physical examination revealed tenderness and percussion pain at the L4–5 level, reduced sensation in the left buttock, posterior and lateral thigh, lateral calf, and dorsum of the foot, positive results on the straight leg raising and strengthening tests of the left lower limb, and bilateral Achilles tendon reflex attenuation. Other examination findings were unremarkable. Lumbar 3D CT identified a posterior marginal separation of the L5 vertebra and a calcified, right-posterior L4–5 intervertebral disc protrusion (Figures 3A,B). Lumbar MRI confirmed L4–5 intervertebral disc herniation and associated spinal canal stenosis (Figures 3C,D). A diagnosis of LDH with spinal stenosis (L4/5) was established. Preoperative VAS and ODI yielded scores of 5/10 and 68, respectively. The patient underwent bilateral transforaminal lumbar interbody fusion (TLIF) and bilateral nerve root exploration and release (Figure 4). Intraoperatively, the posterior marginal osteophyte of the L5 vertebra was observed to displace the L5 traversing nerve root outward into the lateral recess. L5 nerve root was completely encased by a narrowed, bone tunnel-like structure at the upper edge of the L5 vertebra. Postoperatively, dorsiflexion weakness in the right lower limb was noted, with MRC grade 3 strength observed in the tibialis anterior and extensor hallucis longus, while other muscle groups retained normal strength (grade 5). At the 3-month follow-up, the strength of the tibialis anterior and extensor hallucis longus improved to MRC grade 5. The VAS score decreased to 0/10, and the ODI score improved markedly to 12, reflecting no pain and minimal disability. The overall outcome was “Excellent”.

## Discussion

Normally, the traversing nerve root traverses downward closely adjacent to the dura mater at the intervertebral space, with the ligamentum flavum separating it from the facet joint. A degree of mobility exists both the nerve root and the dura mater. During TLIF procedures, removal of the lamina and the facet joint may cause damage to the exiting nerve root, which typically spares the traversing nerve root from injury (10, 11). In this study, preoperative lumbar 3D CT scans and intraoperative observations revealed a right-posterior protrusion of the intervertebral disc with calcification, displacing the traversing nerve root outward into the lateral recess. At the upper edge of the lower vertebral body, the traversing nerve root was entirely encased within a bony tunnel formed by osteophytes. This tunnel-like structure comprised the vertebral posterior margin, the calcified and protruding intervertebral disc, the medial pedicular margin of the lower vertebral body, and a portion of the facet joint, resulting in significant nerve root compression. The traversing nerve root adheres firmly to the osseous structure and traverses within the bony tunnel. Prolonged compression often leads to nerve root edema. Due to the nerve root's limited mobility, alterations in its anatomical positioning and heightened tension significantly increase the risk of inadvertent injury during TLIF procedures involving removal of the facet joint. During TLIF, the traversing nerve root must be retracted medially along with the dural sac to facilitate discectomy and implantation of the fusion cage. Inadequate decompression of the encased traversing nerve root, compounded by its restricted mobility within the bony tunnel, can result in traction-induced nerve root injury during surgical manipulation.

The author thinks that the appropriate surgical strategy involves the following key considerations: 1) A comprehensive preoperative analysis of imaging is imperative. When LDH is accompanied by vertebral posterior marginal osteophytes completely encasing the nerve root, the conventional TLIF procedure is unsuitable. Specifically, instead of *en bloc* removal of the facet joint, an ultrasonic osteotome should be employed to meticulously decompress the dorsal aspect of the bony tunnel surrounding the nerve root. This is followed by sequential and precise decompression of the medial, lateral, and ventral aspects of the traversing nerve root, thereby accomplishing the process of “surrounding nerve root decompression”; 2) Prior to discectomy and fusion cage implantation, the traversing nerve root must be carefully assessed. Even after achieving complete decompression, the mobility of the nerve root may remain restricted. To mitigate traction injury during procedures of discectomy and fusion cage implantation, forceful retraction of the traversing nerve root toward the midline should be avoided as much possible. Whenever feasible, the fusion cage could be implanted from the contralateral side to minimize the risk of nerve root injury; 3) During TLIF procedures, when encountering heightened nerve root tension where retraction poses a risk of iatrogenic injury, a traction-free technique may be employed. This involves performing bilateral facetectomy and placing a smaller interbody cage with contralateral compression to minimize nerve root retraction; 4) Case 2 predominantly exhibited neurological deficits in the left lower limb, yet lumbar 3D CT revealed complete encasement of right traversing L5 nerve root by the osteophyte. Despite the absence or mild presence of symptoms in the right lower limb, right L5 traversing nerve root decompression is necessary due to its immobilization within the bony tunnel. Post-decompression and fusion cage implantation on the left side, the resulting increase in intervertebral space height can exacerbate tension on the right nerve root, potentially inducing severe neurological complications; 5) Conventional TLIF surgery typically involves dorsal decompression of the traversing nerve root. However, in cases where the vertebral posterior marginal osteophyte entirely encases the nerve root, comprehensive exploration and decompression must encompass the dorsal, medial, lateral, and ventral aspects in a systematic approach. Lumbar 3D CT analysis from both cases indicates that the bony tunnel encasing the nerve root extends posteriorly and superiorly to the lower vertebral body, necessitating a sufficiently extensive downward range of nerve root exploration, decompression and release.

To our knowledge, current literature lacks documentation of surgical management for vertebral posterior marginal osteophytes completely encasing the nerve root. This report presents the first documented cases of LDH with such osteophytes, detailing surgical interventions and providing insights to mitigate irreversible nerve root injury in future cases. For such complex ventral pathologies, transforaminal endoscopic lumbar discectomy (TELD) represents an exceptional but viable ventral surgical option (12). Advances in minimally invasive endoscopy, along with innovations in navigation, robotics, and related technologies, make it possible to execute surgical management to dispose of the condition of vertebral posterior marginal osteophytes completely encasing the nerve root under spinal endoscopy (13, 14). These advancements promise more precise and less invasive procedures, though further investigation into specific surgical techniques and approaches remains necessary.

Nevertheless, this study is subject to several limitations: (1) In both cases, intraoperative photographs capturing the positional relationship between the traversing nerve root and the vertebral posterior marginal osteophyte were not preserved, nor were gross images of the encased nerve root being entirely decompressed, which is regrettable; (2) Despite the meticulous surgical techniques employed, both patients experienced varying degrees of postoperative nerve root injury symptoms. However, marked recovery of these symptoms was observed at the 3-month follow-up, likely attributable to prolonged nerve root compression by tunnel-like osteophytes and ischemia-reperfusion injury following complete decompression (15); (3) The limited representativeness of case studies underscores the necessity of larger-sample investigations to validate the findings presented in this study.

## Conclusion

The presence of vertebral posterior marginal osteophytes completely encasing the nerve root represents a notable risk factor for nerve root injury in LDH cases. Tailored surgical approaches are imperative to minimize complications and optimize patient outcomes.

## Data Availability

The original contributions presented in the study are included in the article/Supplementary Material, further inquiries can be directed to the corresponding author.
